# Determination of the molecular basis for coprogen import by Gram-negative bacteria

**DOI:** 10.1107/S2052252519002926

**Published:** 2019-04-05

**Authors:** Rhys Grinter, Trevor Lithgow

**Affiliations:** a Infection and Immunity Program, Biomedicine Discovery Institute and Department of Microbiology, Monash University, Monash, Victoria 3800, Australia; b School of Biology, Monash University, Monash, Victoria 3800, Australia; c Institute of Microbiology and Infection, School of Immunity and Infection, University of Birmingham, Birmingham B15 2TT, England

**Keywords:** siderophores, coprogen, iron piracy, membrane transport, Gram-negative bacteria, structure determination, transporters, membrane proteins, protein structure, X-ray crystallography

## Abstract

In order to survive in mixed microbial communities, some species of fungi secrete coprogens, siderophores that facilitate capture of the scarce nutrient iron. The TonB-dependent transporter FhuE is integrated in the outer membrane of Gram-negative bacteria and has been reported to scavenge these fungally produced coprogens. In this work, an *Escherichia coli* strain was engineered that is dependent solely on FhuE for its access to siderophore-sequestered iron. Using this tool, it is shown that while FhuE is highly active in the import of coprogens, it has some level of promiscuity, acting as a low-affinity transporter for related siderophores. The crystal structure of FhuE in complex with coprogen was determined, providing a structural basis to explain this selective promiscuity. The structural data, in combination with functional analysis, presented in this work show that FhuE has evolved to specifically engage with planar siderophores. A potential evolutionary driver, and a critical consequence of this selectivity, is that it allows FhuE to exclude antibiotics that mimic nonplanar hydroxamate siderophores: these toxic molecules could otherwise cross the outer membrane barrier through a Trojan horse mechanism.

## Abstract 2

This work presents the crystal structure and functional characterization of FhuE, the bacterial outer membrane transporter responsible for the import of coprogen. Coprogen is an iron-scavenging compound produced by fungi, and FhuE allows bacteria to engage in iron piracy from fungal competitors.

## 1. Introduction   

The incredible diversity and abundance of microbial life leads to fierce competition for resources in any given environment (Hibbing *et al.*, 2010 ▸; Fredrickson & Stephanopoulos, 1981 ▸). In this battle for growth and survival, bacteria and fungi deploy a myriad of strategies to kill or hinder their competitors and to seize scarce nutrients required for growth (Ghoul & Mitri, 2016 ▸). One such nutrient is iron: while abundant on Earth, iron is biologically scarce owing to its insolubility under oxygenic conditions (Wandersman & Delepelaire, 2004 ▸). Iron is an essential nutrient for microorganisms that is needed as a cofactor in proteins responsible for electron transport (Cassat & Skaar, 2013 ▸; Troxell *et al.*, 2012 ▸). In order to obtain iron from the environment, the vast majority of bacteria and fungi secrete small molecules termed siderophores. Siderophores are multidentate ligands that bind iron(III) with an exceedingly high affinity, giving them the ability to capture and coordinate iron in a highly stable complex (Guerinot, 1994 ▸). This iron-loaded complex is then recaptured by the microorganism and processed to release the iron for use by the cell (Ferguson & Deisenhofer, 2004 ▸). Siderophore recapture is mediated by cell-surface membrane-transport proteins, which have specificity for a single or a related group of siderophores (Braun & Hantke, 2011 ▸; Schalk *et al.*, 2012 ▸; Hickman *et al.*, 2017 ▸).

**Figure 1:**
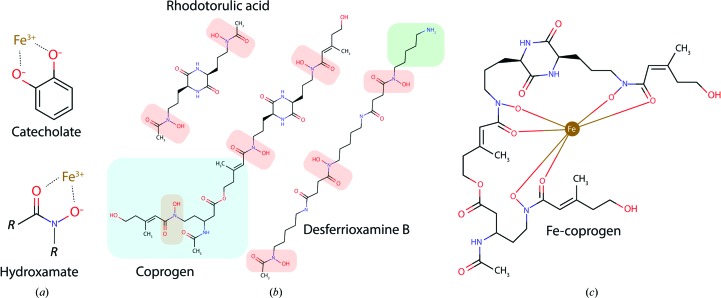
The coprogens: hydroxamate siderophores produced by fungi. (*a*) Bidentate coordination of Fe^3+^ by the siderophore catecholate and hydroxamate functional groups. (*b*) Hydroxamate siderophores produced by fungi (rhodotorulic acid and coprogen) and bacteria (desferrioxamine B). Hydroxamate functional groups are highlighted in red, the fusarinine group of coprogen in aqua and the dangling group of desferrioxamine B in green. (*c*) A two-dimensional representation of Fe^3+^ coordination by coprogen.

While the overall diversity of siderophore molecules is high, with >260 chemically distinct siderophores described to date (Johnstone & Nolan, 2015 ▸), the chemistry of iron-coordinating functional groups is more constrained. Iron(III) is a hard acid and favours interactions with the hard base oxygen (Renshaw *et al.*, 2002 ▸). As such, siderophore functional groups utilize O atoms in a bidentate configuration for iron coordination (Codd, 2008 ▸; Renshaw *et al.*, 2002 ▸). The two most common functional groups are the catecholate and hydroxamate moieties [Fig. 1 ▸(*a*)], with the former being more commonly produced by bacteria and the latter being more common in siderophores of fungal origin (Johnstone & Nolan, 2015 ▸; Codd, 2008 ▸).

The coprogens are a class of linear dihydroxamate or trihydroxamate siderophores that are widely produced by fungi (Hossain *et al.*, 1987 ▸; Hesseltine *et al.*, 1952 ▸). Dihydroxamate coprogens, such as rhodotorulic acid, are formed by the head-to-head condensation of the nonproteinogenic amino acid *N*-acetyl-*N*-hydroxy-l-ornithine, creating a 2,5-diketopiperazine ring that typifies the family [Fig. 1 ▸(*b*); Renshaw *et al.*, 2002 ▸]. In trihydroxamate members of the family, a third hydroxamate moiety is provided by the addition of fusarinine to rhodotorulic acid to create true coprogen [Fig. 1 ▸(*b*); Renshaw *et al.*, 2002 ▸]. Despite containing a 2,5-diketopiperazine ring and side-group modifications, coprogens are linear in structure. Other linear hydroxamate siderophores include ferrioxamine B and ferrioxamine D, which are produced by bacteria such as *Streptomyces pilosus* [Fig. 1 ▸(*b*); Dhungana *et al.*, 2001 ▸; Müller & Raymond, 1984 ▸]. As iron(III) favours an octahedral coordination geometry, three hydroxamate moieties are required to fully coordinate it (Raymond & Carrano, 1979 ▸). Thus, while trihydroxamate coprogens adopt a 1:1 stoichiometry with iron [Fig. 1 ▸(*c*)], the dihydroxamate rhodotorulic acid forms a 3:2 complex of siderophore to iron at physiological pH and a 1:1 complex at pH < 3 (Carrano & Raymond, 1978 ▸). Diversity between different members of the coprogen family is provided by the modification of specific acyl groups to incorporate carboxyl, alkyl and saccharide moieties (Krasnoff *et al.*, 2014 ▸; Renshaw *et al.*, 2002 ▸). These provide the various coprogens with unique chemical properties that can be selectively recognized by integral membrane transporters of the ARN/SIT family responsible for coprogen transport. ARN/SIT transporters belong to a structurally conserved transporter family with 14 α-helical transmembrane spans and are embedded in the plasma membrane of coprogen-secreting fungal species (Haas *et al.*, 2008 ▸; Dias & Sá-Correia, 2013 ▸). The selection pressure drives towards a monopoly of importing the iron-loaded siderophore at the expense of other organisms (Hibbing *et al.*, 2010 ▸; Renshaw *et al.*, 2002 ▸).

When produced in significant quantities, siderophores provide a further competitive advantage to the secreting microbe by inhibiting the growth of other organisms by sequestering environmental iron from them (Winkelmann, 2007 ▸; Traxler *et al.*, 2012 ▸). In order to counteract this general strategy and to engage in siderophore piracy, many microorganisms possess siderophore transporters not only for the molecules that they produce but also for those produced by other microorganisms (Barber & Elde, 2015 ▸; Haas *et al.*, 2008 ▸). For example, FhuE is a transporter in Gram-negative bacteria that can import iron-loaded fungal coprogen (Hantke, 1983 ▸). FhuE belongs to the TonB-dependent transporter (TBDT) family and has been shown to be both necessary and sufficient for the utilization of fungally produced coprogens by *Escherichia coli* and other Gram-negative bacteria (Sauer *et al.*, 1990 ▸; Bitter *et al.*, 1994 ▸; Cui *et al.*, 2006 ▸). While the role of FhuE in coprogen utilization was established some 35 years ago (Hantke, 1983 ▸), the structural and biochemical basis of how FhuE mediates iron piracy has remained unknown.

In this work, we engineered *E. coli* to require FhuE-transported substrates as the sole source of iron for growth. This experimental system demonstrated that while FhuE is highly efficient in the import of coprogens it is also promiscuous, with the ability to import a subset of chemically distinct hydroxamate siderophores. To determine the structural and biochemical basis for its selectivity and transport activity, we solved the crystal structure of FhuE in complex with coprogen. These data show that FhuE employs a selectivity filter in its substrate-binding site, making it specific for planar hydroxamate siderophores. As the ability to control and sequester iron is at the heart of microbial competition (Hibbing *et al.*, 2010 ▸), this work provides significant insight into the molecular mechanisms of microbial iron competition and piracy.

## 2. Materials and methods   

### 2.1. Engineering of the *E. coli* ΔTDBT strain   

The *E. coli* BW25113 ΔTDBT strain was created using the λ Red system (Datsenko & Wanner, 2000 ▸). Kanamycin-resistance cassettes flanked by 300 bp of genomic DNA corresponding to regions in each of the genes encoding the TBDTs of interest were amplified using specific mutants from the *E. coli* Keio collection (Baba *et al.*, 2006 ▸) as templates. Primers are summarized in Supplementary Table S4.

The host strain *E. coli* BW25113 was transformed with the λ Red recombinase plasmid pKD46 (Datsenko & Wanner, 2000 ▸) and grown at 30°C (LB broth + 100 µg ml^−1^ ampicillin) to an OD_600 nm_ of 0.1 before λ Red recombinase was induced by the addition of 0.2% l-arabinose. The cultures were then grown at 30°C until an OD_600 nm_ of 0.6–0.8 was attained and were transformed using the room-temperature electroporation method (Tu *et al.*, 2016 ▸). Briefly, bacterial cells were isolated by centrifugation at 3000*g* for 3 min and washed twice with a volume of sterile 10% glycerol equal to the volume of culture used. The cells were then resuspended in 10% glycerol to a volume of 1/15 of that of the culture. PCR-amplified DNA (100–500 ng) corresponding to the Kan^R^ KO cassette for the gene of interest was then added to 100 µl of the resuspended bacteria and the mixture was electroporated. 1 ml of LB broth was added to the cells post-electroporation, and the culture was recovered at 37°C for 1 h before plating onto LB agar + 30 µg ml^−1^ kanamycin. PCR was used to validate that colonies did indeed have the Kan^R^ cassette in place of the gene of interest.

To remove the Kan^R^ cassette, the mutant strains were transformed with the plasmid pCP20 (Doublet *et al.*, 2008 ▸) containing the ‘flippase cassette’. Cells were grown under either ampicillin (100 µg ml^−1^) or chloramphenicol (30 µg ml^−1^) selection to maintain the plasmid. To remove the Kan^R^ cassette, a single colony of the mutant pCP20-containing strain was used to inoculate 1 ml LB broth (no selection). The culture was grown overnight at 43°C to activate expression of the flippase gene. This culture was then subjected to tenfold serial dilution in sterile LB and plated onto LB agar with no selection. The resulting colonies were patched onto LB agar containing kanamycin, chloramphenicol or no selection. PCR was used to validate that colonies that were unable to grow in the presence of kanamycin or chloramphenicol, but that grew in the absence of selection, were successful for removal of the Kan^R^ cassette. This process was repeated sequentially to derive strains that were multiply defective in up to six TBDT receptors. The order of deletion was *ΔfhuA*, *ΔfecA*, *ΔcirA*, *ΔfepA*, *ΔfhuE* and then *Δfiu*. The mutant strains created in the process were designated Δ1, Δ2, Δ3, Δ4, Δ5 and ΔTBDT based on the number of receptors that had been deleted.

### 2.2. Complementation of *E. coli* ΔTBDT with FhuE and mutant versions of FhuE   

The complete open reading frame for FhuE, including the signal sequence, was amplified from *E. coli* BW25113 by PCR (oligonucleotide primer sequences are given in Supplementary Table S4) and cloned into the plasmid pBAD24 at the EcoRI and HindIII restriction sites. The resulting vector, designated pBADFhuECom, was then transformed into the *E. coli* BW25113 ΔTBDT strain. The plasmid was maintained using 100 µg ml^−1^ ampicillin for selection. In order to assess complementation, *E. coli* BW25113 ΔTBDT pBADFhuECom was streaked onto LB agar with 0.05% arabinose, 100 µg ml^−1^ ampicillin and 50 µ*M* bipyridine spotted with 2 µl of the hydroxamate siderophore. Plates were incubated at room temperature (∼24°C) for 3–7 days and the zone of growth around the siderophore spot was assessed compared with that of the control strain *E. coli* BW25113 ΔTBDT containing the parent plasmid pBAD24.

To assess the relative importance of the amino acids interacting with coprogen in the crystal structure of FhuE, we mutated the residues involved in coprogen binding to alanine using whole-plasmid mutagenesis with pBADFhuECom as the starting vector (Reikofski & Tao, 1992 ▸). Single mutations were introduced using the oligonucleotide primers in Supplementary Table S4. The following double mutants were also generated: R117A/R142A, R117A/W275A and R142A/W275A. This was achieved through a second round of mutagenesis of the pBADFhuECom plasmid using the appropriate primers (Supplementary Table S4). The sequence of the pBADFhuECom template and the introduction of the specified mutations in the resultant plasmids were confirmed by Sanger sequencing. Mutant plasmids were transformed into the *E. coli* BW25113 ΔTBDT strain and tested for function as described for pBADFhuECom.

### 2.3. Protein expression and purification   

For protein production, the FhuE signal sequence was removed by a cloning strategy using the oligonucleotide primers outlined in Supplementary Table S4. DNA was amplified from *E. coli* BW25113 to incorporate NcoI and XhoI restriction sites for cloning into a modified pET-20b vector with an N-terminal 10×His tag followed by a TEV cleavage site. This ligation reaction generated p20bFhuEExp. The resulting plasmid was transformed into *E. coli* BL21 (DE3) C41 cells. After culture in Terrific Broth (12 g tryptone, 24 g yeast extract, 12.26 g K_2_HPO_4_, 2.31 g KH_2_PO_4_ and 4 g glycerol per litre with 100 µg ml^−1^ ampicillin for plasmid selection) at 37°C until an OD_600_ of 1.0 was reached, protein expression was induced with 0.3 m*M* IPTG. The bacteria were cultured for a further 4–5 h at 37°C. Cells were harvested by centrifugation and lysed using a cell disruptor (Emulsiflex) in lysis buffer (50 m*M* Tris, 200 m*M* NaCl, 2 m*M* MgCl_2_) plus 0.1 mg ml^−1^ lysozyme, 0.05 mg ml^−1^ DNAse I and cOmplete Protease-Inhibitor Cocktail Inhibitor tablets (Roche). The resulting lysate was clarified by centrifugation at 10 000*g* for 10 min and the supernatant was then centrifuged for 1 h at 160 000*g* to isolate a membrane fraction. The supernatant was decanted and the membrane pellet was suspended in lysis buffer using a tight-fitting homogenizer. The resuspended membranes were solubilized by the addition of 10% Elugent (Santa Cruz Biotechnology) and incubation with gentle stirring at room temperature for 20 min. The solubilized membrane-protein fraction was clarified by centrifugation at 20 000*g* for 10 min. The supernatant containing the solubilized proteins was applied onto Ni–agarose resin equilibrated in Ni-binding buffer [50 m*M* Tris, 500 m*M* NaCl, 20 m*M* imidazole, 0.03% dodecylmaltoside (DDM) pH 7.9]. The resin was washed with 10–20 column volumes of Ni-binding buffer before elution of the protein with a step gradient of 10%, 25%, 50% and 100% Ni-gradient buffer (50 m*M* Tris, 500 m*M* NaCl, 1 *M* imidazole, 0.03% DDM pH 7.9). FhuE eluted at the 50% and 100% gradient steps. Eluted fractions containing FhuE were pooled and applied onto a 26/600 Superdex S200 size-exclusion column equilibrated with SEC buffer (50 m*M* Tris, 200 m*M* NaCl, 0.03% DDM pH 7.9). To exchange FhuE into the detergent octyl β-d-glucopyranoside (βOG) for crystallo­graphic and biochemical analysis, fractions containing FhuE were pooled and applied onto Ni–agarose resin equilibrated in βOG buffer (50 m*M* Tris, 200 m*M* NaCl, 0.8% βOG pH 7.9). The resin was washed with ten column volumes of βOG buffer before elution with βOG buffer plus 250 m*M* imidazole. Fractions containing FhuE were pooled, and 6×His-tagged TEV protease (final concentration 1 mg ml^−1^) and DTT (final concentration 1 m*M*) were added. This solution was then dialyzed against βOG buffer for 4–6 h at 20°C to allow TEV cleavage of the 10×His tag from FhuE and the removal of excess imidazole. The sample was then applied onto Ni–agarose resin to remove TEV protease and the cleaved decahistidine peptide. The flowthrough containing FhuE from this step was collected, concentrated to 10 mg ml^−1^ in a 30 kDa cutoff centrifugal concentrator, snap-frozen and stored at −80°C.

For control experiments, Fiu was cloned, expressed and purified using the same method as described for FhuE (the primers are listed in Supplementary Table S4).

### 2.4. FhuE substrate-binding analysis by tryptophan fluorescence quenching   

Purified FhuE or Fiu was diluted to 300 n*M* in DDM buffer (50 m*M* Tris, 200 m*M* NaCl, 0.03% DDM pH 7.9) and incubated with hydroxamate siderophores (0–200 µ*M*): Fe-coprogen, Fe-rhodotorulic acid, ferrioxamine B, ferrioxamine E and ferrichrome (obtained from EMC microcollections or from Sigma). The fluorescence of the resulting solutions was measured at 330 and 350 n*M* using a Prometheus NT.48 DSF (NanoTemper) to determine the quenching of binding-site tryptophan residues at a given ligand concentration. Each titration series was performed in triplicate and averaged, with the normalized fluorescence intensity plotted for each compound to obtain substrate-binding curves. Raw data were normalized using the formula *F*_0_/*F* − *F*_0_, where *F*_0_ is the initial fluorescence in the absence of substrate and *F* is the fluorescence observed at a given concentration of ligand.

Thermal stabilization of FhuE in the presence of hydroxamate siderophores was also measured using the Prometheus NT.48 DSF. Thermal melting was performed from 20 to 90°C for FhuE in the presence of siderophores at the concentrations listed above.

### 2.5. Protein crystallization, data collection and structure solution   

Purified FhuE in βOG buffer was screened for crystallization in the presence and absence of its substrate coprogen using commercially available crystallization screens (approximately 600 conditions). For co-crystallization, coprogen (EMC microcollections) was added to purified FhuE, giving final concentrations of 1.25 m*M* coprogen and ∼100 µ*M* FhuE (8 mg ml^−1^).

Crystals formed in numerous conditions in the presence of coprogen, and also in some conditions in the absence of coprogen. Crystals that formed in the presence of coprogen were slightly orange in colour, consistent with the formation of the FhuE–coprogen complex *in crystallo*. A number of conditions were selected for optimization screens; however, these crystals suffered from poor morphology and crystals were therefore harvested directly from the screening trays. Crystals were looped, excess mother liquor was removed by wicking and crystals were flash-cooled to 100 K in liquid N_2_. Crystals were screened for diffraction on the MX2 beamline at the Australian Synchrotron.

Crystals in the absence of coprogen diffracted poorly. However, crystals in the presence of coprogen from a number of conditions diffracted to better than 3 Å resolution. The best diffracting crystal came from the PACT screen and was grown in 0.1 *M* PCB pH 4 (buffer), 25%(*w*/*v*) PEG 1500 (Newman *et al.*, 2005 ▸). This crystal suffered from significant anisotropy but diffracted to at least 2 Å resolution along the a* and b* axes. Multiple wedges of data were collected from different sites of this crystal, processed with *XDS*, scaled using *XSCALE* and merged with *AIMLESS* from the *CCP*4 package (Kabsch, 2010 ▸; Winn *et al.*, 2011 ▸); data were collected to a maximum resolution of 2.00 Å at the detector edge and the resolution was limited to 2.00 Å during processing. Data output from *XSCALE* were elliptically truncated and scaled anisotropically using the Diffraction Anisotropy Server (Strong *et al.*, 2006 ▸). Reflections in outer resolution shells with an *F*/σ of <3.0 (the average *F*/σ of discarded reflections was 2.55) were discarded, leading to resolution limits of 2.00, 2.10 and 3.20 Å along a*, b* and c*, respectively. Anisotropic scale factors were then applied to the data set, followed by an isotropic *B* factor of −41.78 Å^2^. The crystal structure was solved in space group *P*2_1_2_1_2_1_ by molecular replacement with *Phaser* (McCoy *et al.*, 2007 ▸) using a model derived from the TDBT FauA from *Bordetella pertussis* (PDB entry 3efm; 37% amino-acid identity; Brillet *et al.*, 2009 ▸) . The model was built and refined using *phenix.refine* from the *PHENIX* package, *AutoBUSTER * and *Coot* (Adams *et al.*, 2010 ▸; Emsley *et al.*, 2010 ▸; Bricogne *et al.*, 2019 ▸). The entire FhuE polypeptide chain was modelled into the available density, with clear electron density for coprogen observed in the extracellular cavity of FhuE. The quality of the refined FhuE–coprogen crystal structure was validated using the *MolProbity* web server (Chen *et al.*, 2010 ▸). For generation of the composite OMIT map, the ‘composite omit map’ tool from the *PHENIX* package was used (Adams *et al.*, 2010 ▸). Maps were calculated using model phases of FhuE built and refined before coprogen was modelled to ensure that there was no residual model bias.

### 2.6. *In silico* docking   

In order to determine potential ligand-binding sites in the crystal structure of FhuE, an *in silico* docking approach was applied using *AutoDock Vina* via the command line and within the *UCSF Chimera* software package (Pettersen *et al.*, 2004 ▸; Trott & Olson, 2010 ▸). The coordinates for ferrioxamine B were obtained from the Cambridge Crystallographic Data Centre (accession code OFUYET; Dhungana *et al.*, 2001 ▸). Coordinates for Fe-rhodotorulic acid in a 3:2 complex with iron in configurations 1 and 2 were built manually in *PyMOL* and *UCSF Chimera* before being subjected to energy-minimization refinement and regularization using the *UCSF Chimera* package (Pettersen *et al.*, 2004 ▸). A box size of 46.5 × 56.0 × 45.5 Å was utilized encompassing the entire extracellular portion of FhuE. A total of 9–10 binding modes were sought for each docking run, with a search exhaustiveness of 8–100 and a maximum energy difference of 3 kcal mol^−1^. Docking solutions were visually inspected and the highest rated solution was used for the main figures and for discussions.

## 3. Results   

### 3.1. FhuE utilizes Fe-coprogen as a high-affinity substrate   

**Figure 2:**
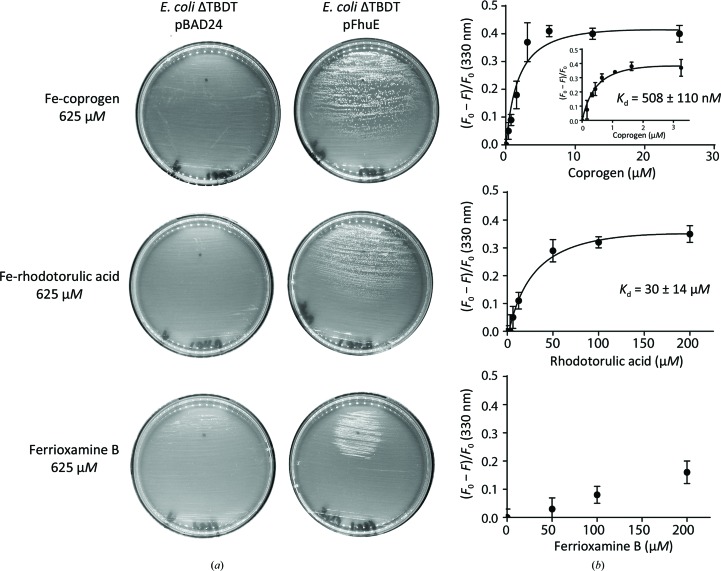
The ability of FhuE to utilize hydroxamate siderophores correlates with *in vitro* binding affinity. (*a*) A solid agar growth assay of the *E. coli* strain lacking other high-affinity uptake systems (*E. coli* ΔTBDT; see Section 2), illustrating FhuE-dependent growth using either coprogen, rhodotorulic acid or ferrioxamine B as an exclusive source of iron. Plates were spotted with the siderophore solution and the growth of bacteria expressing FhuE (+pFhuE) or not (+pBAD24) was assessed after incubation for 72 h at 24°C. (*b*) Plot of normalized fluorescence quenching of FhuE at 330 nm at increasing concentrations of siderophore. For Fe-coprogen and Fe-rhodotorulic acid, the observed change in fluorescence is attributable to the quenching of tryptophan residues in the FhuE substrate-binding pocket owing to siderophore binding. *F*_0_, fluorescence at 0 µ*M* substrate concentration; *F*, fluorescence at the listed substrate concentration. Error bars are derived from the standard deviation of three independent experiments.

Previous reports on FhuE (Hantke, 1983 ▸; Matzanke *et al.*, 1984 ▸) suggested that it can import three linear hydroxamate siderophores, coprogen, rhodotorulic acid and ferrioxamine B (Fig. 1 ▸), but not the cyclic siderophores ferrioxamine E or ferrichrome. However, the relative affinity of FhuE for these substrates was unclear. Measuring the specificity of FhuE for these substrates *in vivo* required a genetically modified biological system. To this end, we engineered a strain of *E. coli* BW25113 that lacks all of the TBDTs involved in iron acquisition (designated ΔTBDT; Section 2). This bacterial strain grows very poorly on solid media without the addition of exogenous iron [Fig. 2 ▸(*a*)]. Transforming the ΔTBDT strain with a plasmid to drive the expression of FhuE supported growth on media containing coprogen, rhodotorulic acid or ferrioxamine B as an iron source, but not those with ferrichrome or ferrioxamine E [Fig. 2 ▸(*a*), Supplementary Fig. S1(*a*)]. Of the three siderophores that supported growth, the extent of growth enhancement differed markedly. Fe-coprogen supported a large zone of growth, Fe-rhodotorulic acid a slightly smaller zone and ferrioxamine B a comparatively small zone, suggesting lower efficiency in the import of the two latter substrates [Fig. 2 ▸(*a*)]. To test this proposition, FhuE was purified and tryptophan fluorescence quenching was used to directly measure its affinity for the siderophores. Quenching of FhuE fluorescence was observed upon titration of all five siderophores. For Fe-coprogen and Fe-rhodotorulic acid this decrease in fluorescence was titratable, giving dis­association constants of 508 ± 116 n*M* for Fe-coprogen and 30 ± 14 µ*M* for Fe-rhodotorulic acid [Fig. 2 ▸(*b*)].

For ferrioxamine B, ferrioxamine E and ferrichrome the degree of observed quenching was variable and did not saturate at a ligand concentration of up to 200 µ*M* [Fig. 2 ▸(*b*), Supplementary Fig. S1(*b*)]. To differentiate tryptophan fluorescence quenching owing to substrate binding from non­specific quenching at high concentrations of substrate, we performed a control experiment with purified Fiu, a non­hydroxamate siderophore receptor, in place of FhuE (Nikaido & Rosenberg, 1990 ▸). For these three siderophores the magnitude of the quenching observed for Fiu was similar to that for FhuE [Supplementary Figs. S1(*a*) and S2], suggesting that the majority of the quenching observed for these compounds is nonspecific and they are bound very weakly by FhuE, if at all. For Fe-coprogen and Fe-rhodotorulic acid this nonspecific quenching was negligible (Supplementary Fig. S2).

For a further independent assessment of its interaction with these siderophores, FhuE was subjected to thermal melting in the presence of various concentrations of substrate. In agreement with the substrate-binding assays and substrate-dependent growth assays, Fe-coprogen and Fe-rhodotorulic acid stabilized FhuE, while ferrioxamine B, ferrichrome and ferrioxamine E did not. Coprogen exhibited the strongest shift in melting temperature: from 69.8 to 72.5°C. As expected, Fiu was not stabilized by any of the siderophores (Supplementary Fig. S3).

Taken together, these data show that while FhuE is capable of binding and transporting Fe-coprogen, Fe-rhodotorulic acid and ferrioxamine B, there is a large difference in substrate affinity and transport efficiency.

### 3.2. The crystal structure of FhuE reveals selectivity for planar siderophores   

**Figure 3:**
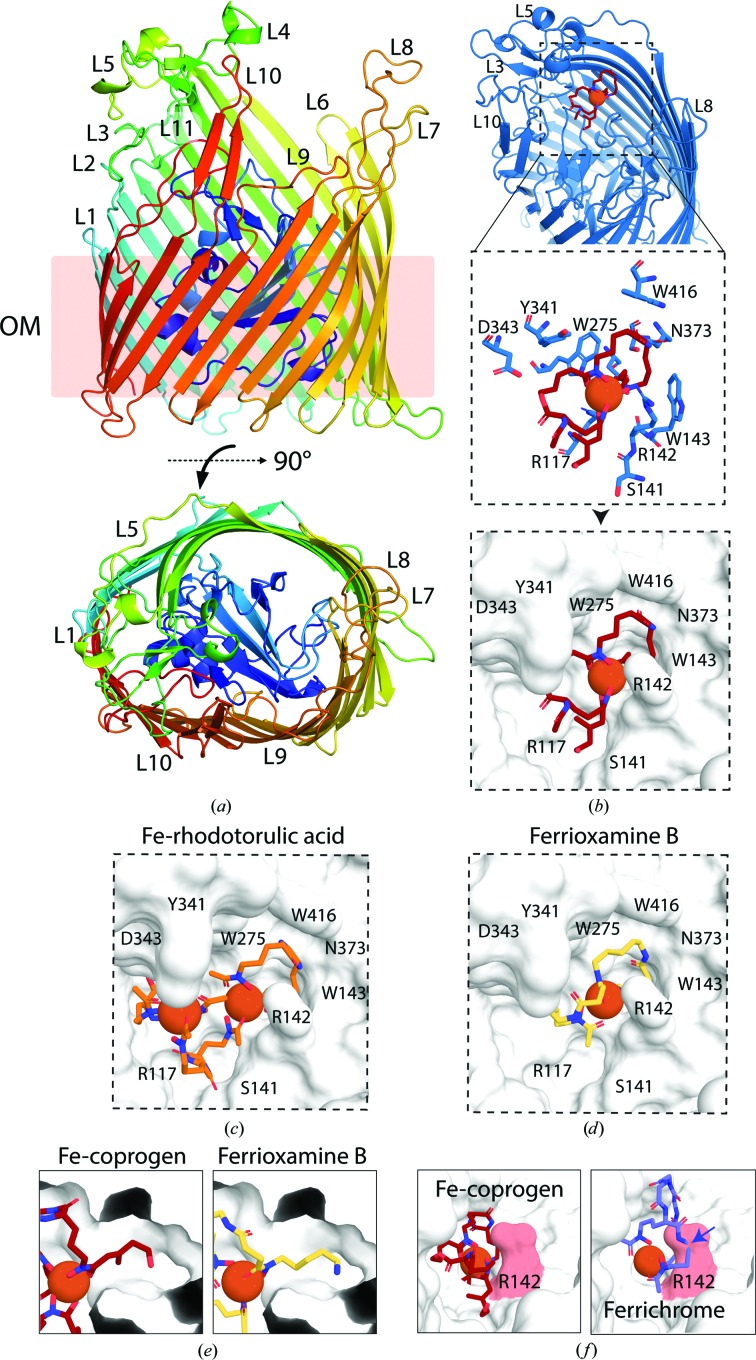
The crystal structure of FhuE in complex with coprogen reveals a substrate-binding site selective for planar hydroxamate siderophores. (*a*) Structure of FhuE coloured as a Jones’ rainbow from the N-terminus (blue) to the C-­terminus (red). The transmembrane region is indicated as being within the outer membrane (OM), and extracellular loops are labelled from L1 to L11. Transposed by 90°, the lower panel shows a top-down view of FhuE. (*b*) Fe-coprogen co-crystallized with FhuE is shown bound to the substrate-binding pocket as a red stick model, with FhuE as a blue cartoon (top), key interacting residues as blue sticks (middle) and a white surface representation (bottom). Models of Fe-rhodotorulic acid (*c*) and ferrioxamine B (*d*) in complex with FhuE derived from *in silico* docking are shown with FhuE as a white surface representation. (*e*) The location of the coprogen *trans*-anhydromevalonyl group in the substrate-binding tunnel of FhuE and the dangling group of ferrioxamine B docked in an analogous position. (*f*) The role of Arg142 in restricting the FhuE binding pocket to planar hydroxamate siderophores. Planar Fe-coprogen is accommodated in the FhuE binding site (left), while the nonplanar sidero­phore ferrichrome is sterically excluded by Arg142 (right).

To determine the molecular basis for the transport of both high-affinity and low-affinity substrates by FhuE, we solved the crystal structure of FhuE in complex with Fe-coprogen (Supplementary Table S1). The structure of FhuE consists of a canonical TBDT fold (Noinaj *et al.*, 2010 ▸) consisting of a 22-stranded transmembrane β-barrel, the lumen of which is occluded by a globular plug domain [Fig. 3 ▸(*a*)]. Comparison of FhuE to other structures in the PDB using the *DALI* server (Holm & Laakso, 2016 ▸) revealed that it is structurally most similar to the ferripyoverdine receptor FpvAI (PDB entry 2iah; *Z*-score of 43.7; r.m.s.d. of 2.0 Å; C. Wirth, W. Meyer-Klaucke, F. Pattus & D. Cobessi, unpublished work) from the bacterium *Pseudomonas aeruginosa* (Supplementary Table S2). FhuE shares a relatively modest 36% amino-acid identity with FpvAI. Pyoverdine, the substrate of FpvAI, is a mixed-ligand siderophore that is widely produced by *Pseudomonas* spp. and is structurally unrelated to coprogen [Supplementary Fig. S4(*a*); Cobessi *et al.*, 2005 ▸]. The most parsimonious explanation for the observed structural homology between these two relatively distantly related transporters is that they originated from a common ancestor and have evolved to bind distinct iron-loaded substrates.

Electron density attributable to coprogen in complex with a single Fe ion was present in an internal cavity in the surface of FhuE, into which Fe-coprogen was modelled [Supplementary Fig. S5(*a*), Supplementary Data]. Coprogen sits in a semi-enclosed binding pocket coordinated by a combination of aromatic, polar and charged residues [Fig. 3 ▸(*b*)]. Notably, all three hydroxamate groups of coprogen are coordinated by FhuE through two arginine residues (Arg117 and Arg142) and a tryptophan residue (Trp275). An additional hydrogen bond is observed between one of the keto O atoms of the coprogen diketopiperazine ring and an asparagine residue (Asn373), with the remainder of the binding pocket being composed of hydrophobic interactions [Fig. 3 ▸(*b*), Supplementary Fig. S5(*b*) and Supplementary Movie S1]. Interestingly, comparative analysis of the structure of FpvAI in complex with pyoverdine revealed that the substrate-binding site, as judged by the location of the coordinated Fe atom, is conserved between FhuE and FpvAI. Residues equivalent to Arg117 and Trp275 were also conserved in FpvAI, where they form interactions with Fe-coordinating pyoverdine functional groups. However, the other residues involved in substrate interactions and the overall shape of the binding pockets are distinct [Supplementary Figs. S4(*b*) and S4(*c*)].

In order to determine the binding mode for the low-affinity FhuE substrates Fe-rhodotorulic acid and ferrioxamine B, we performed *in silico* docking with the FhuE crystal structure (Supplementary Table S3; Dhungana *et al.*, 2001 ▸). Previous work showed that at physiological pH rhodotorulic acid adopts a 3:2 complex with iron (Carrano & Raymond, 1978 ▸). In this work, the authors proposed a model for this complex in which three rhodotorulic acid molecules bridge between two Fe ions to form a complex with threefold pseudosymmetry [Supplementary Fig. S6(*a*)]. However, this complex coordinates Fe in a nonplanar configuration which appears to be incompatible with the FhuE substrate-binding site. In order to satisfy the observed 3:2 stoichiometry, an alternative possibility exists in which each Fe atom is coordinated by two hydroxamate groups from a single rhodotorulic acid molecule, with the third rhodotorulic acid molecule bridging two of these complexes through a single interaction with each Fe atom [Supplementary Fig. S6(*b*)]. As the structure of Fe-rhodotorulic acid has not been determined experimentally, we prepared models of each of these two possible configurations and performed docking with FhuE. Fe-rhodotorulic acid in configuration 1 did not dock into the substrate-binding site identified in the FhuE–coprogen crystal structure [Supplementary Fig. S6(*c*)]. However, configuration 2 docked with the FhuE substrate-binding site with one rhodotorulic acid molecule from the complex in an analogous position to Fe-coprogen, with the coordinated Fe atoms within 0.4 Å [Fig. 3 ▸(*c*), Supplementary Fig. S6(*a*), Supplementary Data]. This result suggests that FhuE is most suited to binding configuration 2 of the Fe-rhodotorulic acid complex. However, we cannot preclude the possibility that flexibility in the FhuE binding site allows accommodation of the originally proposed conformation 1 (Carrano & Raymond, 1978 ▸). Further experimental evidence is required to determine the nature of iron coordination by this siderophore.

For ferrioxamine B the most favoured docking solution also placed this molecule in an analogous position to that of Fe-coprogen. The Fe atoms in the complexes are within 0.2 Å, and the loops formed through the coordination of iron are in a similar planar orientation [Fig. 3 ▸(*d*)]. The dangling chain of ferrioxamine B, which terminates in an amine group, penetrates a narrow cavity in the FhuE binding pocket [Fig. 1 ▸(*b*)]. In the crystal structure this cavity accommodates the *trans*-anhydro­­mevalonyl group of coprogen in the analogous position [Fig. 3 ▸(*e*)]. This docking result suggests that the binding mode between Fe-coprogen and ferrioxamine B is analogous, raising the prospect that the large difference in affinity observed between the two molecules can be attributed to tailoring of the FhuE binding pocket to Fe-coprogen through the course of evolution.

The crystal structure of FhuE also provides an explanation as to why it is unable to utilize the trihydroxamate siderophore ferrichrome as a substrate (Hantke, 1983 ▸). The planar nature of the FhuE binding pocket does not allow the coordination of the Fe-ferrichrome complex without significant clashes. The direct coordination via Arg142 of the hydroxamate groups appears to act as a selectivity filter, only allowing Fe-hydroxamate complexes in a planar configuration to be accommodated in the substrate-binding pocket [Figs. 3 ▸(*b*), 3 ▸(*c*) and 3 ▸(*d*)]. Superimposition of the nonplanar ferrichrome with coprogen shows significant clashes with Arg142 [Fig. 3 ▸(*e*), Supplementary Movie S2].

### 3.3. Functional mutagenesis of FhuE shows that hydroxamate coordination is crucial for substrate import   

**Figure 4:**
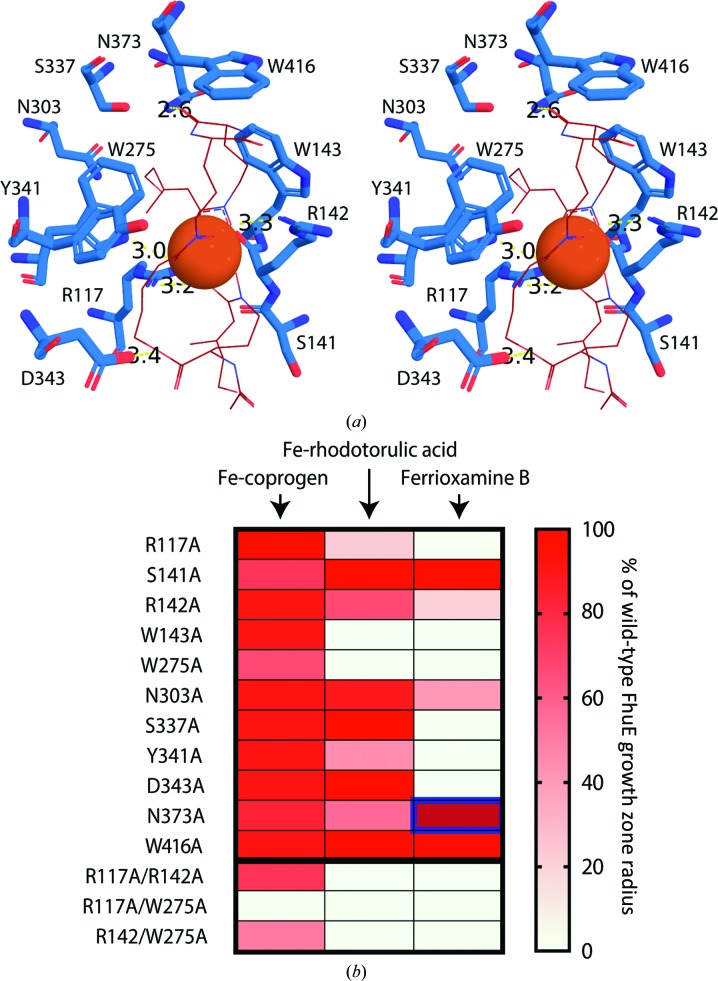
Mutagenesis of the FhuE substrate-binding site reveals residues that are key for receptor function and selectivity. (*a*) A cross-eyed stereo representation of the FhuE substrate-binding site containing Fe-coprogen. Residues involved in coprogen coordination that were subjected to mutagenesis are shown as blue sticks, coprogen is shown as a red wire and Fe is shown as an orange sphere. Interactions between FhuE and coprogen of <3.5 Å are labelled. (*b*) The effect of mutations of FhuE on its ability to utilize the siderophores as an iron source to support the growth of *E. coli* ΔTBDT on solid agar. Growth differences were scored by observing the size of growth around a spot of siderophore compared with that of wild-type FhuE. The growth of the N373A mutant with ferrioxamine B as a substrate is highlighted; using this substrate, the growth of the N373A mutant exceeded that of wild-type FhuE.

To address the role of the amino acids that constitute the FhuE substrate-binding pocket, we mutated all residues directly involved in coordinating Fe-coprogen to alanine [Fig. 4 ▸(*a*)]. The *E. coli* ΔTBDT strain was used to express these mutant variants of FhuE, and the ability of the FhuE variants to support growth at varying siderophore concentrations was assessed [Fig. 4 ▸(*b*), Supplementary Fig. S7]. Double mutants were also constructed for R117A, R142A and W275A that directly coordinate the hydroxamate moieties of Fe-coprogen. All of the mutants were then scored for fitness compared with the strain expressing ‘wild-type’ FhuE.

Each of the single mutations yielded a relatively minor effect on the ability of FhuE to utilize coprogen, but all double mutants were impaired in their activity, with the R117A/W275A mutant being totally nonfunctional [Fig. 4 ▸(*b*), Supplementary Fig. S7]. It is possible that expression levels differ between mutant and wild-type FhuE, leading to a difference in the growth phenotype in this assay. However, none of the amino acids that were mutated play a structural role in FhuE and the TBDT fold is highly stable, making it less likely that these mutations would have a major effect on FhuE expression. Based on this assumption, these data show that the enclosed binding site of FhuE and the large number of residues involved in coordinating Fe-coprogen mean that any individual mutation of the binding site can be tolerated. However, the mutation of multiple residues interacting with the iron-loaded coprogen is deleterious for substrate acquisition. Interestingly, while Trp275 and Arg117 are crucial for Fe-coprogen utilization, Arg142 apparently plays a more minor role in the process. This supports the notion of a distinct role for Arg142 in substrate selection by defining the planar FhuE binding pocket.

A distinct situation was in evidence when assessing the role of mutant variants of FhuE in utilizing Fe-rhodotorulic acid as a substrate. Single mutations had a more major effect, with the W143A and W275A mutants and the R117A/R142A double mutant failing to grow on Fe-rhodotorulic acid as an iron source and with the R117A, Y341A and N373A mutants leading to a pronounced reduction in substrate utilization [Fig. 4 ▸(*b*), Supplementary Fig. S7]. Thus, while the same set of residues do provide for utilization of iron-loaded coprogen for cell growth, rhodotorulic acid utilization is selectively defective. We suggest that this is likely to reflect the smaller number of binding-site residues involved in interaction with this substrate [Fig. 3 ▸(*c*)] and the observed weaker binding affinity [Fig. 2 ▸(*b*)]. As expected, the residues Ser141, Asn303, Ser337 and Asp343 in the binding site that form interactions with Fe-coprogen but are unlikely to coordinate Fe-rhodotorulic acid did not affect the function of FhuE with this substrate (Supplementary Movie S3).

### 3.4. Mutation of Asn373 to alanine in FhuE leads to enhanced ferrioxamine B import   

**Figure 5:**
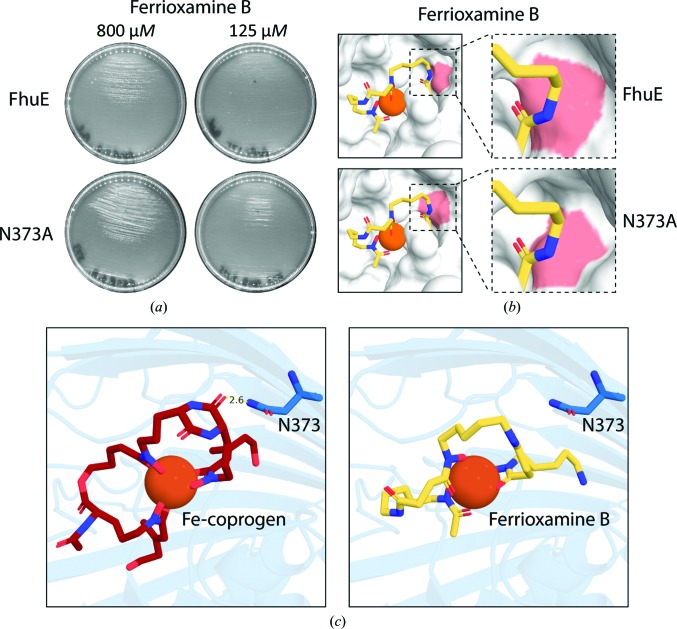
Mutation of Asn373 enhances the ability of FhuE to utilize ferrioxamine B. (*a*) A solid agar growth assay showing the enhanced ability of the N373A mutant to support growth using ferrioxamine B as an iron source relative to the growth observed in the presence of FhuE. (*b*) Structural modelling of the N373A mutant with ferrioxamine B docked into the substrate-binding pocket. The removal of the asparagine side chain opens the substrate-binding channel of FhuE to better accommodate ferrioxamine B. (*c*) The position of Asn373 (blue sticks) relative to coprogen (left) and ferrioxamine B (right). In the crystal structure, Asn373 forms a hydrogen bond to a carbonyl group from the coprogen diketopiperazine ring. This group is not present in ferrioxamine B.

The majority of the mutations had a severe effect on the ability of FhuE to utilize ferrioxamine B as an iron source [Fig. 4 ▸(*b*), Supplementary Movie S3], with only S141A, N373A and W416A mutants permitting significant FhuE-mediated growth using ferrioxamine B. This observation is consistent with the very weak binding observed between FhuE and ferrioxamine B [Fig. 2 ▸(*b*)]. Strikingly, rather than decreasing the ability of FhuE to utilize ferrioxamine, the N373A mutation led to a significant increase in growth [Fig. 5 ▸(*a*), Supplementary Figure S7]. Analysis of the structure of FhuE with docked ferrioxamine B provides a molecular basis for this improved utilization: Asn373 forms one side of the channel entrance in FhuE, with the N373A mutation opening up this region, better accommodating the dangling chain of ferrioxamine B [Figs. 5 ▸(*b*) and 5 ▸(*c*), Supplementary Movie S4]. This experimental system thereby provides insight into how the random acquisition of mutations in nature could allow the modification of FhuE substrate specificity. Mutations of this kind could represent evolutionary tinkering, allowing bacteria to evolve transporters with novel substrate specificity, as indicated by the structural relationships observed between FhuE and the ferripyoverdine receptor FpvAI from the bacterium *P. aeruginosa* (Supplementary Table S1 and Fig. S4).

## 4. Discussion   

Biologically available iron is a limiting resource in polymicrobial communities. There is a growing awareness that some bacteria employ a ‘Black Queen’ strategy in which they utilize resources produced by the community rather than producing their own. This strategy leads to the loss of genes encoding the production of these resources and to the reliance on other community members to satisfy their requirements (Morris *et al.*, 2012 ▸). Applying this strategy, bacteria utilize iron sequestered in siderophores made by other members of the community as their source of this nutrient rather than producing their own siderophores (Ratledge & Dover, 2000 ▸; Morris, 2015 ▸). The high affinity and selectivity of FhuE for coprogen siderophores that we report in this study provides an advantage to the producing bacteria. In a mixed microbial community FhuE would allow the producing bacteria to acquire iron sequestered in these fungal siderophores at a far lower selective cost than producing their own (Khan *et al.*, 2006 ▸; Hibbing *et al.*, 2010 ▸).

This lower cost of siderophore ‘piracy’ explains the existence of FhuE, but does not explain its observed specificity for coprogen and other planar siderophores. Why did FhuE not evolve a substrate-binding site that could accommodate both planar and nonplanar hydroxamate siderophores? This strategy is adopted by the periplasmic siderophore-binding protein FhuD: this soluble protein acts downstream of TBDTs and binds diverse hydroxamate siderophores including Fe-coprogen, ferrioxamine B and ferrichrome. FhuD mediates this generalist binding through an open binding pocket, which interacts with its siderophore binding partner via direct interactions with the hydroxamate–iron complex (Supplementary Fig. S8, Supplementary Movie S5; Clarke *et al.*, 2002 ▸).

In part, this question is answered by the need to ensure sufficiently high-affinity binding to Fe-coprogen to compete effectively against the siderophore-producing species. However, nutritional need is not the only evolutionary force at play in microbial communities. In addition to their function in siderophore import, TBDTs are also the target of antimicrobial toxins. Such molecules are composed of an antibiotic activity fused to a siderophore, or molecules that have evolved to mimic the TBDT substrate (Górska *et al.*, 2014 ▸; Ferguson *et al.*, 2001 ▸; Grinter *et al.*, 2014 ▸, 2016 ▸). An example of the former is albomycin, an antibiotic ferrichrome fusion that binds to, and is imported by, the ferrichrome transporter FhuA (Ferguson *et al.*, 2000 ▸). Under conditions in which albomycin is present, FhuA represents a liability to the bacterium. The specificity of FhuE for planar siderophores excludes not only ferrichrome, as we demonstrate in this study, but also ferrichrome–antibiotic fusions such as albomycin. In a scenario where albomycin is present, the exclusion of nonplanar siderophores by FhuE prevents antibiotic import, while allowing FhuE to continue to function in iron acquisition via Fe-coprogen. Understood from this perspective, the lack of a single generic receptor for hydroxamate siderophore import in Gram-negative bacteria makes sense; it represents an evolutionary bet-hedging strategy, which counteracts the targeting of TBDTs by antimicrobial siderophore mimics.

While FhuE can import ferrioxamine B, it has a low affinity for the substrate and it therefore provides a relatively modest level of growth. However, we show that the mutation N373A in FhuE enhances its ability to import ferrioxamine B. This mutation would provide a clear advantage to bacteria growing in a community where ferrioxamine B was present by increasing their ability to utilize this siderophore as an iron source. In this scenario, if the presence of ferrioxamine B was sustained it would lead to the selection of additional mutations in FhuE that enhance the utilization of this substrate, and over time to a receptor with a new specificity range. These evolutionary forces are likely to be echoed in the observed similarities between the substrate-binding sites of FhuE and FpvAI, which share key binding residues despite the fact they transport highly structurally distinct substrates (Schauer *et al.*, 2008 ▸). TBDTs share a common fold and a deep evolutionary history, but have diverged radically in their amino-acid compositions and have evolved distinct binding sites to target different substrates. This process, which is observed in miniature in this work, has allowed bacterial species to cope with new ecological challenges.

## Supplementary Material

PDB reference: FhuE in complex with its substrate coprogen, 6e4v

Supplementary Movie S1. DOI: 10.1107/S2052252519002926/jt5033sup1.mp4

Supplementary Movie S2. DOI: 10.1107/S2052252519002926/jt5033sup2.mp4

Supplementary Movie S3. DOI: 10.1107/S2052252519002926/jt5033sup3.mp4

Supplementary Movie S4. DOI: 10.1107/S2052252519002926/jt5033sup4.mp4

Supplementary Movie S5. DOI: 10.1107/S2052252519002926/jt5033sup5.mp4

Supplementary Movie captions, Supplementary Data. Supplementary Figures and Supplementary Tables S1, S2 and S3. DOI: 10.1107/S2052252519002926/jt5033sup6.pdf

Supplementary Table S4. DOI: 10.1107/S2052252519002926/jt5033sup7.xlsx

Supplementary Table S5. DOI: 10.1107/S2052252519002926/jt5033sup8.xlsx

PDBQT file for ferrioxamine B. DOI: 10.1107/S2052252519002926/jt5033sup9.txt

PDBQT file for FhuE. DOI: 10.1107/S2052252519002926/jt5033sup10.txt

PDBQT file for Fe-rhodotorulic acid in configuration 1. DOI: 10.1107/S2052252519002926/jt5033sup11.txt

PDBQT file for Fe-rhodotorulic acid in configuration 2. DOI: 10.1107/S2052252519002926/jt5033sup12.txt
