# Supplementary material for: Determination of the molecular basis for coprogen import by Gram-negative bacteria
Source: IUCrJ. 2019 Apr 5;6(Pt 3):401–11. doi: 10.1107/S2052252519002926 (PMC6503915; doi:10.1107/S2052252519002926)
Supplement: Supplementary file 6 [file m-06-00401-sup6.pdf]

# IUCrJ

**Volume 6 (2019)**


**Supporting information for article:**

## Determination of the molecular basis for coprogen import by Gram-negative bacteria

**Rhys Grinter and Trevor Lithgow**

## S1. Supplemental Movies

**Movie S1** The crystal structure of FhuE in complex with coprogen. The transmembrane region of FhuE is delineated with yellow spheres, the coloring scheme for structural components is the same as figure 1.

**Movie S2** Arginine 142 (R142) in FhuE constitutes a selectivity filter for planar hydroxamate siderophores. FhuE is shown as a white surface representation, coprogen is a red stick model, and ferrichrome is a lilac stick model. R142 is highlighted in pink. This movie, shows that ferrichrome is excluded from the FhuE binding pocket by arginine 142, due to its non-planar configuration. Mutation of this residue to alanine removes this clash

**Movie S3** Mapping the effect of FhuE binding site mutations of the ability to utilize rhodotorulic acid and ferrioxamine B. Rhodotorulic acid is shown as orange sticks, amino acids in the FhuE substrate binding pocket are shown concurrently as sticks and color coded based on the effect of their mutation of rhodotorulic acid utilization (white for complete loss of function, pink for partial loss of function and red for no observed loss of function). Ferrioxamine B is shown after rhodotorulic acid as a yellow stick model with the same visualization parameters for the effect of mutations on utilization.

**Movie S4** Mutation of FhuE asparagine 373 (N373) to alanine widens the substrate binding channel. FhuE is shown as a white surface, with N373 highlighted in pink, ferrioxamine B is shown as a yellow stick model. This movie illustrates that mutation of asparagine 373 to alanine opens the FhuE substrate binding channel which potentially makes it better able to accommodate the ferrioxamine B dangling chain. This observation potentially explains the enhanced ability of FhuE N373A to utilize ferrioxamine B as an iron source.

**Movie S5** The periplasmic binding protein FhuD binds diverse hydroxamate siderophores. R84 and Y106 of FhuD interact with analogous hydroxamate groups of Fe-coprogen (PDB ID = 1ESZ), ferrioxamine B (PDB ID = 1K2V) and the ferrichrome analogue albomycin (PDB ID = 1K7S). These residues interact with the siderophore in an open binding pocket, which allows for accommodation of siderophores with diverse backbones and planarity 16. Coloring for this animation is the same as figure S8.

## S2. Supporting Data

PDBQT files for *in silico* docking experiments have been deposited as supporting data.

jt5033sup9.txt is that for ferrioxamine B.

jt5033sup10.txt is that for FhuE.

jt5033sup11.txt is that for Fe-rhodotorulic acid in configuration 1.

jt5033sup12.txt is that for Fe-rhodotorulic acid in configuration 2.

**Table S1** Crystallography data collection and refinement statistics for FhuE

| | **FhuE-Coprogen Complex** |
|---|---|
| **Data Collection Statistics (all data)**[a] | |
| Wavelength (Å) | 0.9763 |
| Space Group | $P2_12_12_1$ |
| Unit cell parameters | |
| *a, b, c* (Å) | 68.13, 103.77, 118.62 |
| *α, β, γ* (°) | 90, 90, 90 |
| Resolution Range (Å) | 47.53 - 2.00 (2.05-2.00) |
| $R_{merge}$ | 59.7 (413.4) |
| $R_{pim}$ | 10.9 (82.5) |
| $I/\sigma(I)$ | 4.7 (0.7) |
| CC(1/2) | 0.958 (0.873) |
| Completeness (%) | 99.9 (99.0) |
| Redundancy | 30.5 (26.1) |
| No. unique reflection | 57586 (4121) |
| **Anisotropic Processing**[b] | |
| Resolution truncation (reflections with F/σ < 3.0 discarded) | |
| *a*, b*, c* (Å)* | 2.00, 2.10, 3.20 |
| Resolution Range (Å) | 47.53 – 2.00 (2.05-2.00) |
| $R_{merge}$ | 37.53 (54.6) |
| $I/\sigma(I)$ | 10.5 (5.2) |
| Completeness (%) | |
| 47.55 to 3.42 Å | 100.0 |
| 3.42 to 2.70 Å | 87.4 |
| 2.70 to 2.00 Å | 36.2 (3.6) |
| Redundancy | 28.96 (25.9) |
| No. unique reflections | 35629 (148) |
| **Refinement statistics** | |
| Resolution range (anisotropically truncated) | 47.53 – 2.00 |
| No. of reflections | 35629 |
| $R_{work}/R_{free}$ | 0.219/0.246 |
| No. atoms | |
| *Protein* | 5319 |
| *Ligand / ions* | 294 |
| R.m.s deviations | |
| Bond lengths (Å) | 0.009 |

| | |
|---|---|
| Bond angles (°) | 1.66 |
| Ramachandran Statistics | |
| Favored, Allowed, Outliers (%) | 97.76, 2.24, 0.00 |

[a] Values in parentheses are for highest-resolution shell.

[b] Correction applied using 'Diffraction Anisotropy Server'

**Table S2** Output from the DALI server showing structures with similarity to FhuE.

| # | PDB ID | Chain | Z | rmsd | lali | nres | %id | Description |
|---|---|---|---|---|---|---|---|---|
| 1 | 5ODW | A | 45.2 | 2.2 | 654 | 772 | 36 | FpvA - Ferripyoverdine transporter |
| 2 | 1XKW | A | 42.6 | 2.2 | 628 | 655 | 31 | FptA - Fe(iii)-pyochelin transporter |
| 3 | 3EFM | A | 42.0 | 1.8 | 552 | 572 | 37 | FauA- Ferric alcaligin siderophore transporter |
| 4 | 3QLB | A | 38.1 | 2.5 | 623 | 673 | 21 | Enantio-pyochelin transporter; |
| 5 | 1FI1 | A | 36.7 | 2.6 | 632 | 707 | 20 | FhuA - Ferrichrome-iron transporter |
| 6 | 6BPM | A | 36.5 | 2.2 | 619 | 711 | 21 | Fiu - Catecholate siderophore transporter |
| 7 | 5FP1 | A | 35.7 | 2.5 | 619 | 701 | 25 | PiuA Pa. - Catecholate siderophore transporter |
| 8 | 6H7F | A | 35.1 | 2.7 | 620 | 674 | 20 | BauA - Ferric preacinetobactin transporter |
| 9 | 5FOK | A | 34.4 | 2.5 | 590 | 656 | 21 | PiuA Ab. - Iron transport outer membrane transporter |
| 10 | 4EPA | A | 33.3 | 2.8 | 566 | 632 | 17 | FyuA - Pesticin transporter |
| 11 | 2HDI | A | 33.2 | 3.2 | 529 | 598 | 19 | Cir - Catecholate siderophore transporter |
| 12 | 2GSK | A | 33.0 | 3.0 | 520 | 590 | 17 | BtuB - Vitamin b12 transporter |
| 13 | 3FHH | A | 32.7 | 3.0 | 551 | 621 | 18 | ShuA - Outer membrane heme transporter |
| 14 | 1KMO | A | 30.7 | 2.8 | 576 | 661 | 16 | FecA -Iron(iii) dicitrate transport protein |
| 15 | 5M9B | A | 29.0 | 3.2 | 548 | 707 | 19 | PfeA Ferric enterobactin transporter |
| 16 | 5FP2 | A | 28.6 | 3.3 | 466 | 499 | 18 | PirA Pa. - Ferric enterobactin transporter |
| 17 | 3V89 | A | 28.1 | 3.9 | 559 | 853 | 14 | TbpA- Transferrin-binding protein a |
| 18 | 3CSL | A | 27.4 | 3.3 | 557 | 753 | 17 | HasR - Hemophore transporter |
| 19 | 4AIP | B | 26.9 | 3.3 | 547 | 665 | 16 | FrpB - Ferric/ferrous iron transporter |
| 20 | 5FR8 | A | 26.5 | 3.1 | 550 | 707 | 17 | SusD - Polysacharide transporter |
| 21 | 5FQ6 | M | 25.7 | 3.9 | 582 | 948 | 14 | SusD - Polysacharide transporter |
| 22 | 4RDR | A | 25.0 | 3.4 | 543 | 706 | 13 | ZnuD - Zinc transporter |
| 23 | 4ZGV | A | 24.7 | 4.1 | 557 | 809 | 13 | FusA - Ferredoxin transporter |
| 24 | 5T4Y | C | 13.4 | 4.9 | 456 | 807 | 13 | SusD-like - Probable peptide transporter |

Pa. = P. aeruginosa, Ab. = Acinetobacter baumannii

**Table S3** Statistics for docking of Fe-rhodotorulic acid and ferrioxamine B performed using AutoDock.

| Mode | Affinity (kcal/mol) | Distance from RMSD l.b. | Best Mode RMSD u.b. |
|---|---|---|---|
| | Fe-rhodotorulic Acid Configuraton 1 | | |
| 1 | -11.8 | 0 | 0 |
| 2 | -11.3 | 1.828 | 3.381 |
| 3 | -11.1 | 1.774 | 5.17 |
| 4 | -10.9 | 6.94 | 10.942 |
| 5 | -10.9 | 6.173 | 10.474 |
| 6 | -10.7 | 6.72 | 10.887 |
| 7 | -10.6 | 3.232 | 6.279 |
| 8 | -10.3 | 3.532 | 6.293 |
| 9 | -10.2 | 12.198 | 18.013 |
| | Fe-rhodotorulic Acid Configuraton 2 | | |
| 1 | -9.9 | 0 | 0 |
| 2 | -9.8 | 1.84 | 10.583 |
| 3 | -9.7 | 2.376 | 10.675 |
| 4 | -9.6 | 9.258 | 14.193 |
| 5 | -9.5 | 1.645 | 6.469 |
| 6 | -9.4 | 2.963 | 10.753 |
| 7 | -9.4 | 2.166 | 6.98 |
| 8 | -9.3 | 3.348 | 11.024 |
| 9 | -9.3 | 10.305 | 14.498 |
| | Ferrioxamine B | | |
| 1 | -9.7 | 0 | 0 |
| 2 | -8.9 | 6.509 | 10.246 |
| 3 | -8.6 | 13.413 | 15.894 |
| 4 | -8.6 | 14.194 | 17.822 |
| 5 | -8.5 | 12.572 | 13.984 |
| 6 | -8.4 | 13.793 | 16.909 |
| 7 | -8.3 | 12.833 | 14.213 |
| 8 | -8.1 | 13.489 | 14.971 |
| 9 | -8.1 | 13.958 | 17.238 |
| 10 | -8 | 5.001 | 7.154 |

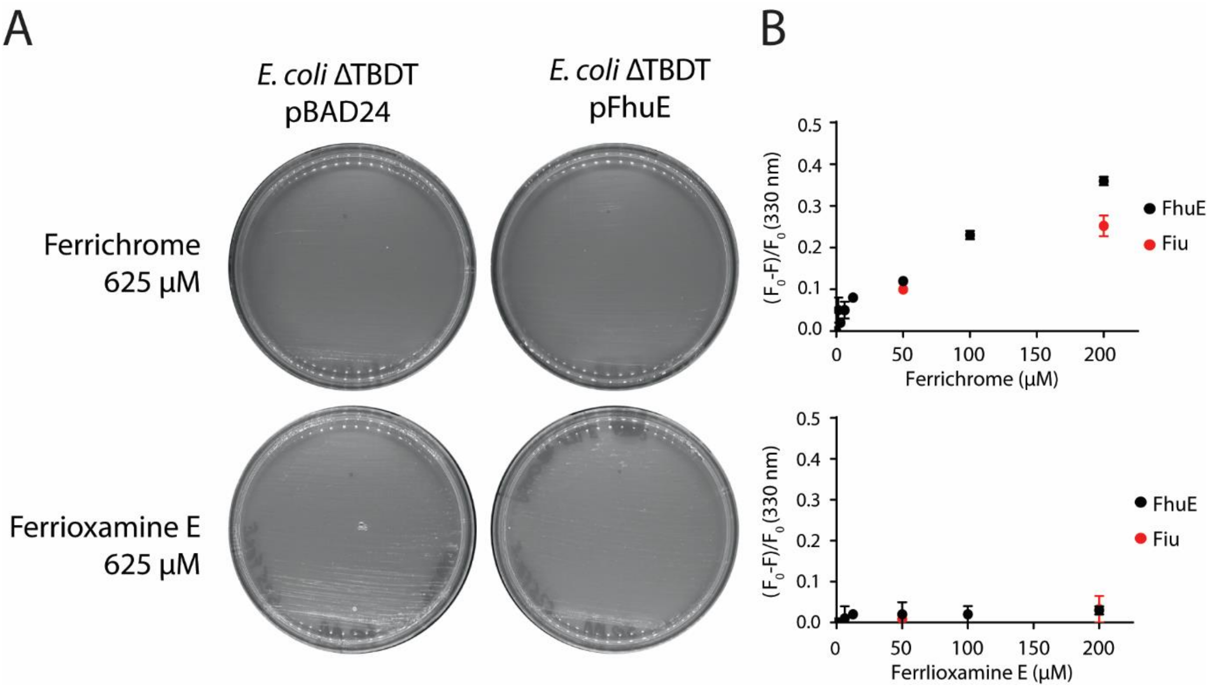


**Figure S1** Ferrichrome and Ferrioxamine E are not substrates for FhuE. (A) A solid agar growth assay of the *E. coli* strain lacking other high affinity uptake systems (*E. coli* ΔTBDT, see Methods), illustrating the inability of FhuE to utilize ferrichrome or ferrioxamine E as a source of iron. Plates were spotted with the siderophore solution and streaked with *E. coli* ΔTBDT containing either pFhuE or the control vector pBAD24 growth was observed after incubation at 24 C for 72 hours. (B) Plot of normalized fluorescence quenching of FhuE and the non-ferrioxamine binding receptor Fiu at 330nm, at increasing concentrations of ferrichrome or ferrioxamine E. The similarity of fluorescence loss between FhuE and Fiu suggests non-specific quenching, rather than binding is occurring with these siderophores. F0 = fluorescence at 0 substrate concentration, F = fluorescence at listed substrate concentration. Error bars are derived from the standard deviation of 3 independent experiments.

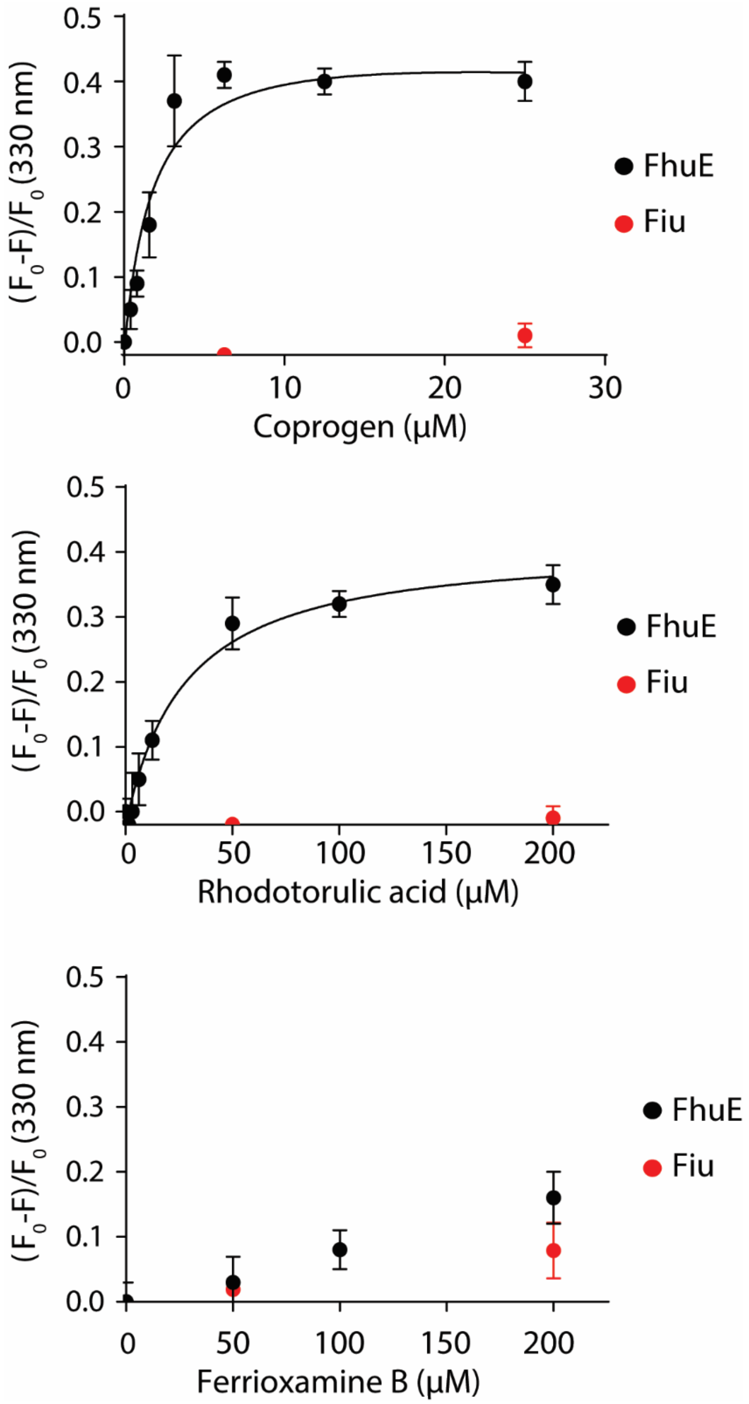


**Figure S2** Control tryptophan fluorescence quenching assays for FhuE transported siderophores. Plot of normalized fluorescence quenching of FhuE and the non-ferrioxamine binding receptor Fiu at 330nm, at increasing concentrations of Fe-coprogen, Fe-rhodotorulic acid and ferrioxamine B. The similarity of fluorescence decrease between FhuE and Fiu with ferrioxamine B suggests non-specific quenching, rather than binding accounts for the majority of fluorescence loss with this substrate. F0 = fluorescence at 0 substrate concentration, F = fluorescence at listed substrate concentration. Error bars are derived from the standard deviation of 3 independent experiments.

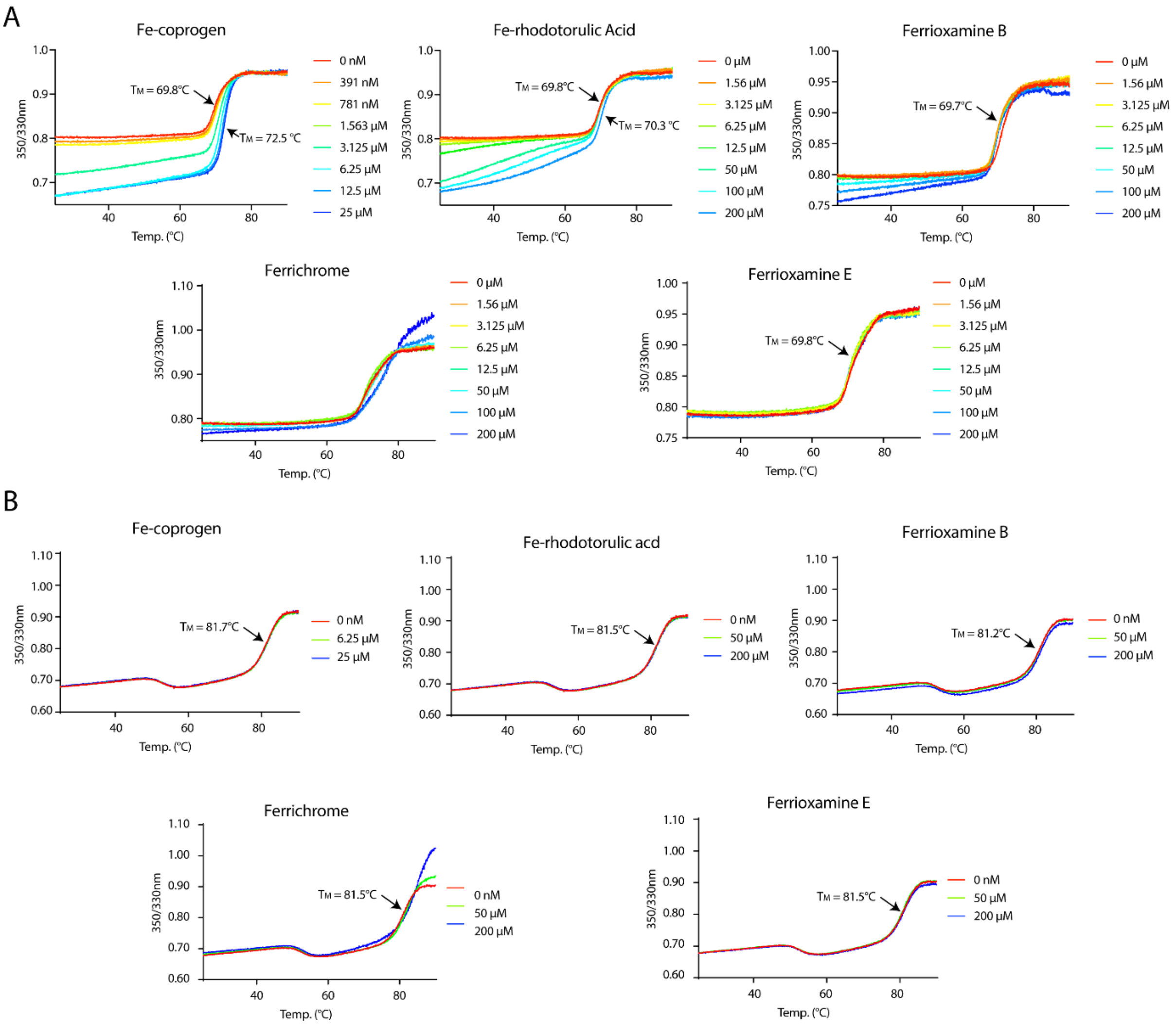


**Figure S3** Thermal melting curves for FhuE and Fiu in the presence of hydroxamate siderophores. Thermal melting curves for coprogen transporter FhuE (A) and the Fe-catecholate transporter Fiu (B) at various concentrations of hydroxamate siderophore. Change in ratio of fluorescence at 350/330 nm was recorded over a melting run of 20 to 90 °C. For ferrichrome a concentration dependent change in the 350/330nm ratio is observed with temperature, however this occurs in both FhuE and the Fiu control and so is unlikely to be related to stabilizing interactions.

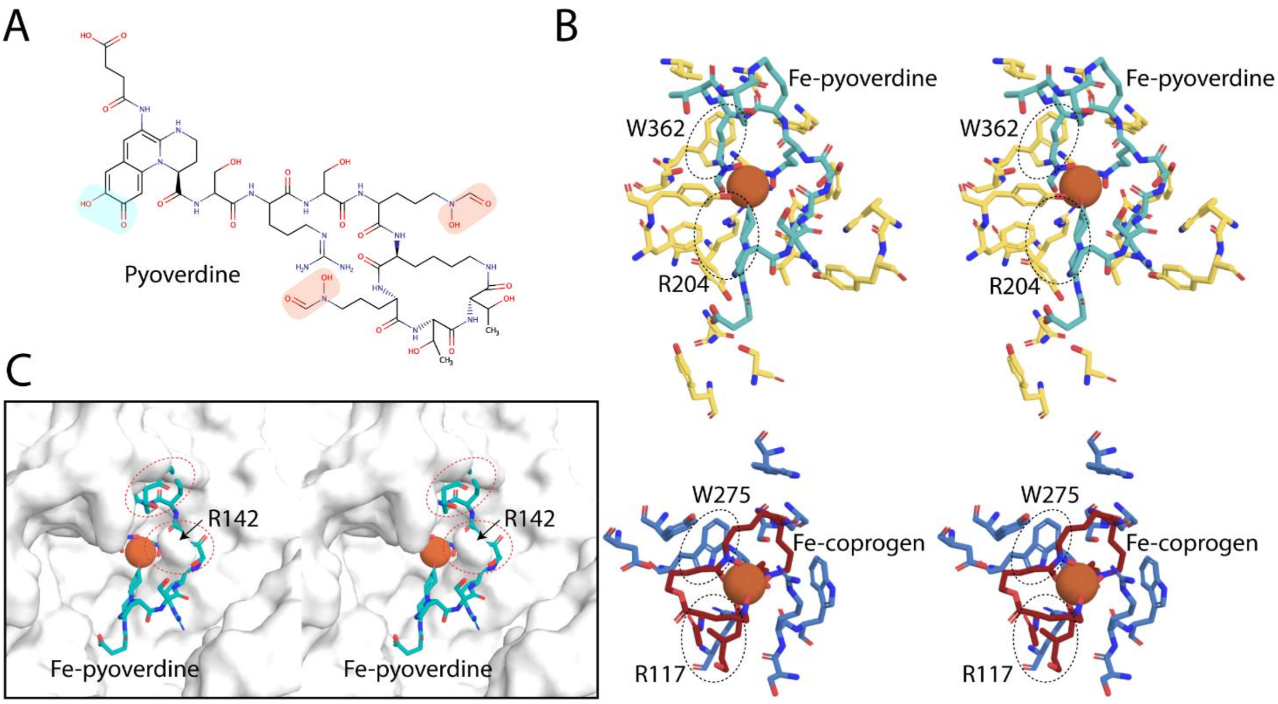


**Figure S4** Comparison of the FpvA and FhuE substrate binding sites. (A) The two-dimensional structure of pyoverdine, the substrate for FpvA from *P. aeruginosa*. Hydroxamate functional groups are highlighted in salmon, the catecholate functional group is highlighted in aqua. (B) Comparison of the FpvA Fe-pyoverdine binding site (top) and FhuE Fe-coprogen binding site (bottom), showing residues involved in coordinating their respective substrates. The arginine and tryptophan residues that are conserved between the two binding sites are labelled and circled. (C) The position of Fe-pyoverdine relative to the substrate binding site of FhuE when FpvA is superimposed, significant clashes between FhuE and Fe-pyoverdine are illustrated with red circles.

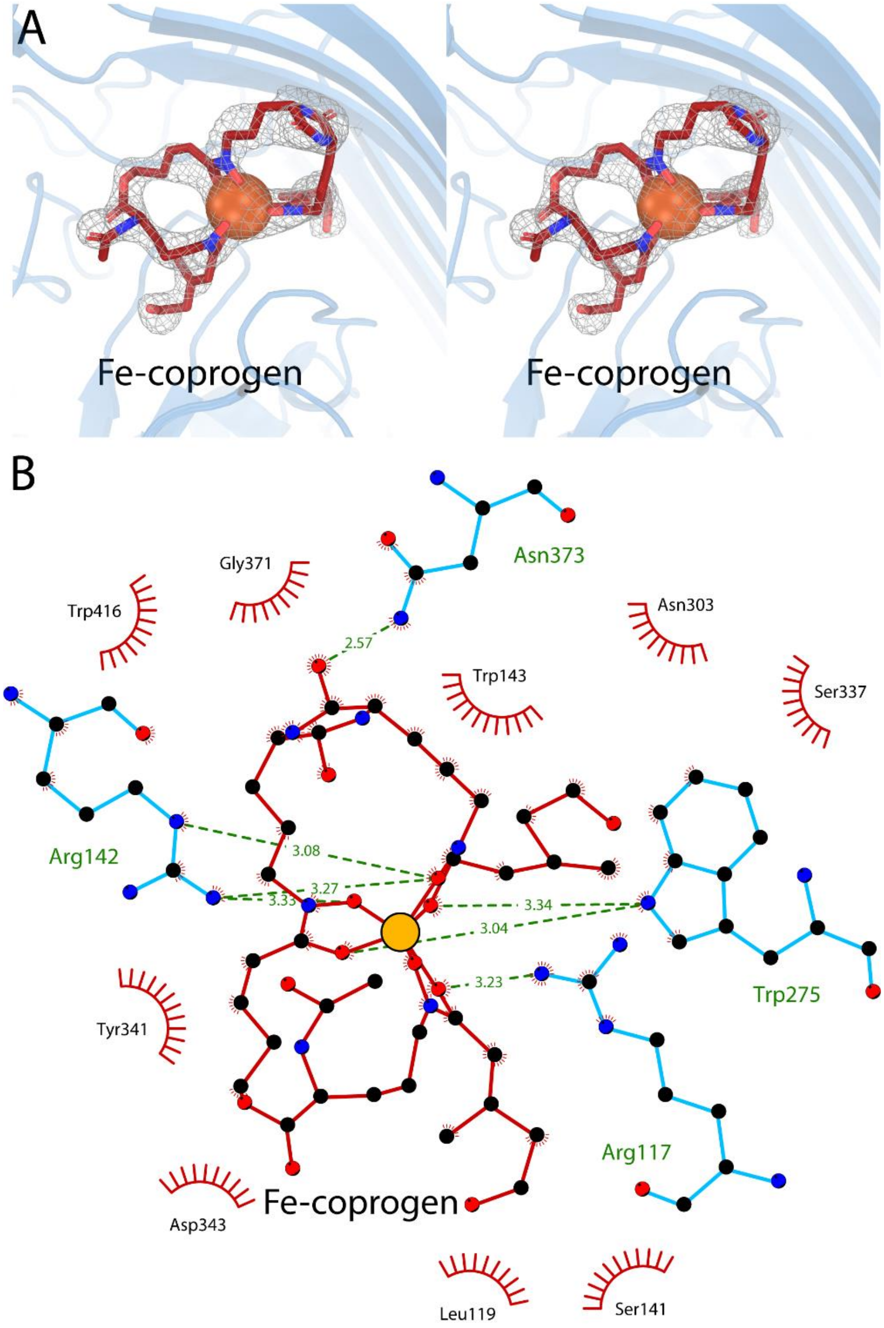


**Figure S5** The coordination and binding of coprogen in the substrate binding pocket of FhuE. (A) A cross-eye stereo view of composite omit map density attributable to coprogen in the FhuE crystal structure. The structure of FhuE is represented in blue, coprogen is shown as red sticks with the Fe atom as an orange sphere. Electron density is shown as a grey wire frame and contoured to 1.0 σ. (B) A 2D representation of interactions between FhuE and coprogen in the FhuE substrate binding pocket. FhuE residues forming polar or charge interactions with coprogen are shown in blue, residues forming non-polar interactions are shown as red starbursts. Coprogen is shown as red sticks with the Fe atom shown as an orange sphere.

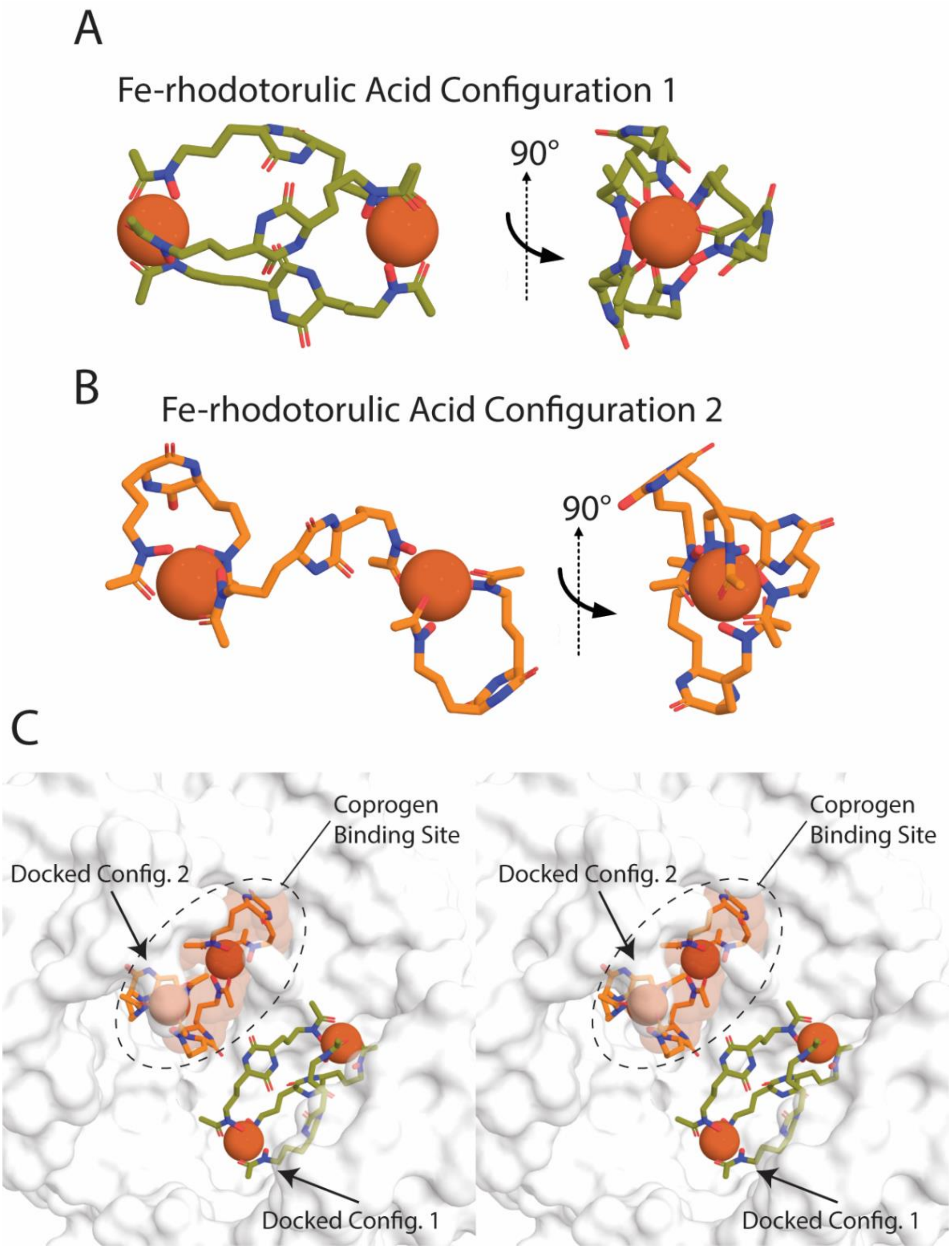


**Figure S6** In silico docking of Fe-rhodotorulic acid with FhuE suggests an previously un-proposed configuration exists in solution. Fe-rhodotorulic acid exists in as a 3:2 complex with Fe3+ at physiological pH 1. Two configurations for the complex are possible with this stoichiometry as shown in panels (A) and (B). In silico docking of models of these complexes with the crystal structure of FhuE, shown as a stereo view in (C), only places the second configuration of Fe-rhodotorulic in the FhuE substrate binding site, suggesting the complex is transported in this configuration by FhuE.

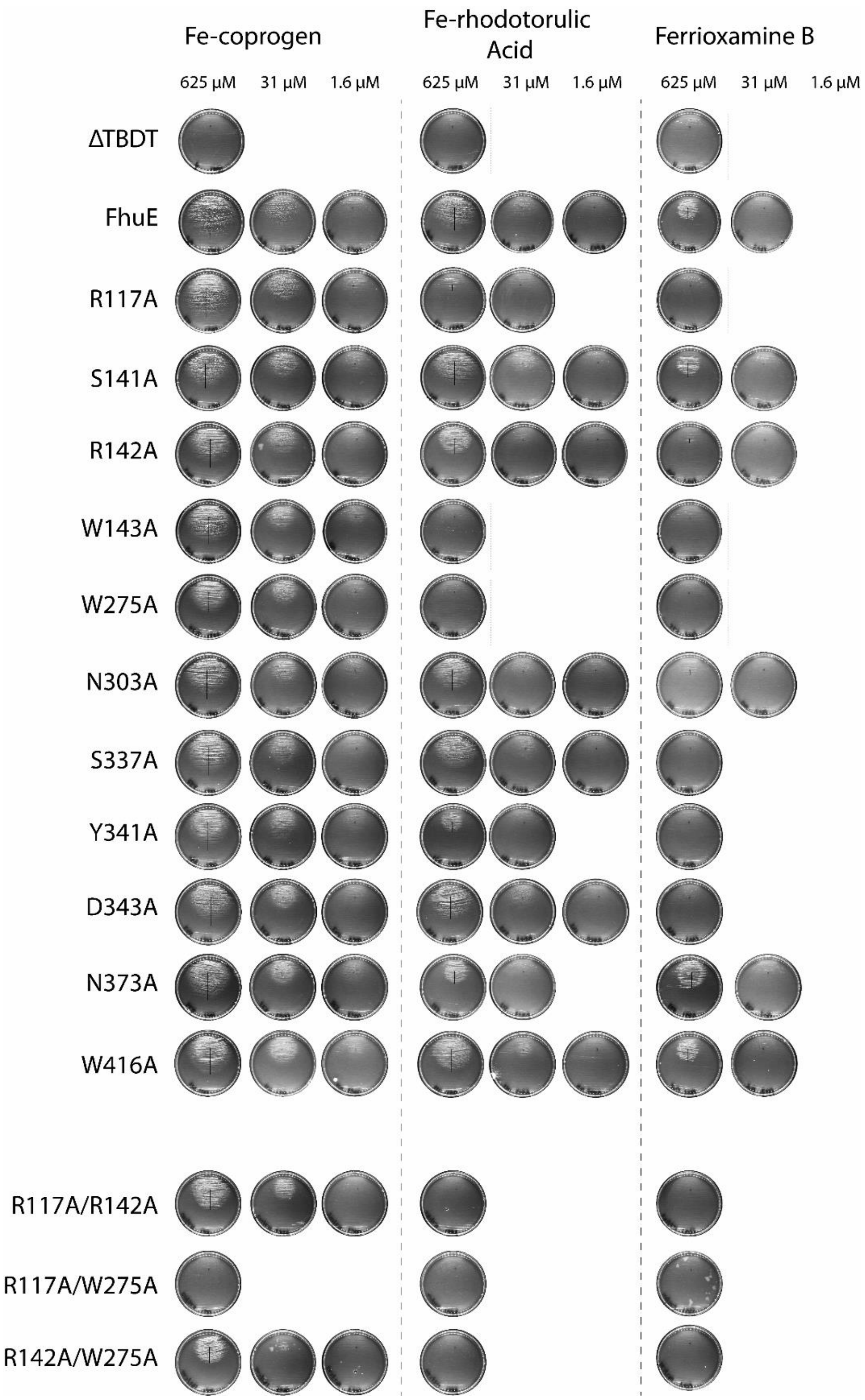


**Figure S7** The ability of FhuE mutants to utilize siderophores as an iron source. The *E. coli* ΔTBDT strain transformed with the various mutant versions of FhuE were incubated in the presence of various concentrations of siderophore. Lines denote the length of the zone of growth away from a spot of siderophore containing solution, the length of these lines was used to assess a growth defect compared to *E. coli* ΔTBDT expressing FhuE.

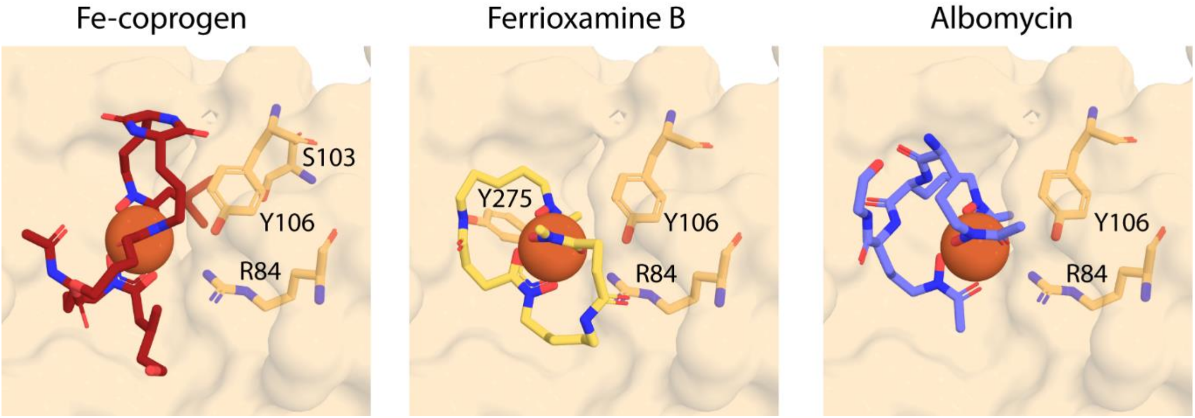


**Figure S8** The periplasmic binding protein FhuD binds diverse hydroxamate siderophores. R84 and Y106 of FhuD interact with analogous hydroxamate groups of Fe-coprogen (PDB ID = 1ESZ), ferrioxamine B (PDB ID = 1K2V) and the ferrichrome analogue albomycin (PDB ID = 1K7S). These residues interact with the siderophore in an open binding pocket, which allows for accommodation of siderophores with diverse backbones and planarity 2.
